# Graphene-Based Nanostructures Produced by Laser Ablation Assisted by Electric Field

**DOI:** 10.3390/nano16010072

**Published:** 2026-01-04

**Authors:** Mariapompea Cutroneo, Vaclav Holy, Petr Malinsky, Petr Slepicka, Alena Michalcova, Lorenzo Torrisi

**Affiliations:** 1 Department of Physics (MIFT), Messina University, V. le F.S. d’Alcontres 31, S. Agata, 98166 Messina, Italy; 2 Department of Condensed Matter Physics, Faculty of Mathematics and Physics, Charles University, Ke Karlovu 5, 121 16 Praha, Czech Republic; 3 Nuclear Physics Institute, AS CR, 250 68 Rez, Czech Republic; 4 Department of Physics, Faculty of Science, University of J. E. Purkyně, Českémládeže 8, 400 96 Ústí nad Labem, Czech Republic; 5 Department of Solid State Engineering, University of Chemistry and Technology Prague, 166 28 Prague, Czech Republic; 6 Department od Metals and Corrosion Engineering, University of Chemistry and Technology Prague 5, 166 28 Prague, Czech Republic

**Keywords:** graphene-based material, nanostructures, laser ablation in liquid, electric field

## Abstract

The properties of carbon-based materials with nanometric size support their use in numerous applications, such as optoelectronics and energy devices, bioimaging, photodetectors, and sensors. Among the various nanostructure fabrication methods, pulsed laser ablation in liquids (PLA) is widely recognized for its simplicity and rapid processing. It is considered an environmentally friendly synthesis, as it enables nanostructure fabrication in pure liquids without chemical reagents, activators, or vacuum systems, in line with the increasing interest in sustainable and green nanotechnologies. A great challenge of PLA is the reproducibility of the size and shape of the produced structure. This can be accomplished by selection of the proper laser parameters and characteristics of the used liquid. This study is focused on the comparison of the synthesis of graphene-based nanostructures by electric-field-assisted pulsed laser ablation of a graphite target immersed in distilled water and deionized water, used as separate liquid media, without the use of chemical reagents. This is an innovative and environmentally friendly approach for the production of graphene nanoparticles. The laser parameters were kept constant throughout the experiments, while different voltage values were applied between the electrodes immersed in the liquid medium. The applied electric field significantly influences plasma dynamics, cavitation bubble evolution, and post-ablation nanoparticle growth processes, enabling controlled tuning of nanoparticle size and morphology. The optical properties of the obtained suspensions were evaluated by UV–Vis and FTIR spectroscopies. Atomic force microscopy revealed the composition, morphology, and quality of the formed structures.

## 1. Introduction

Since 2010, when Andre Geim and Konstantin Novoselov were awarded the Nobel Prize, numerous studies have been conducted on graphene in view of its unique properties, deeply different from its bulk structure [1].

Graphene consists of carbon atoms bonded in a honeycomb lattice, one-atom-thick, forming a 2D structure. Among the remarkable features of graphene are its high transparency [2], high electron mobility of about 2.5 × 10^5^ cm^2^ V^−1^s^−1^ at room temperature [3], high thermal conductivity of about 3000 Wm^−1^K^−1^ [4], and the possibility to be chemically functionalized [5]. A recent fascinating study by Cao et al. [6] revealed the twofold nature of graphene bilayers, which can be tuned from an insulator to a superconductor depending on the angle formed by twisting the two graphene sheets relative to each other. Although the electronic, optical, thermal, and mechanical properties of graphene and its derivatives have gathered the attention of the scientific community, an undeniable challenge is the ability to scale graphene up for commercial use. This has motivated the development of experimental techniques for reproducible manufacturing of graphene as large sheets with good quality, without defects, and single-crystal. The thickness of the graphene sheets is crucial, as single-layer graphene displayed high carrier mobilities [7], bi-layers revealed variable band gap [8], and multi-layers demonstrated high ohmic behavior.

The production of graphene has a relatively long tradition, ranging from top-down to bottom-up approaches. The mechanical exfoliation of graphite yields high-quality graphene, but it is unsuitable for large-scale production. Chemical vapor deposition (CVD) and epitaxial growth on a substrate [9,10,11] enable large-area graphene films, but they require expensive apparatus and complex processing steps, and this can be a limit in terms of cost-effectiveness when large quantities of graphene are required for industrial applications.

Liquid-phase exfoliation (LPE) is a promising approach for scalable production of graphene, although it requires high energy to separate layers in the bulk material, introducing defects and limiting the control of size, thickness, and defect density as well as the production of large, few-layer sheets [12]. As an alternative, laser-based techniques have been widely investigated. Typically, lasers are used for fundamental physics research [13], for applications in the biomedical field [14], and for materials modification [15]. Typically, during laser-generated plasma in a vacuum of metallic targets irradiated by pulsed lasers [15], high-intensity charge separation effects with emission of ions accelerated mostly along the normal to the target surface are induced [16]. The properties of the target (electrical, optical, roughness, thickness) strongly affect the penetration depth of the laser, the absorption of laser energy, and the electron density of the generated plasma. An important parameter affecting the laser–matter interaction is the temporal width [17] of the laser pulse. When a nanosecond pulse irradiates a solid target, it causes ablation, melting, evaporation, and ionization. A femtosecond laser causes ablation of the material via sublimation; that is, the solid material is transformed into vapor. A picosecond pulse can be considered an intermediate case with respect to the previous two. A crucial parameter in laser–matter interaction is the laser fluence, defined as the energy per unit area (J/cm^2^). Focusing the laser through a lens concentrates energy onto a smaller area, affecting ablation efficiency and nanoparticle production. Obviously, a laser beam passing through a lens is focused to a smaller area, and consequently, all the energy of the beam is focused into an area smaller than the initial diameter of the laser beam [18]. It is worth noting that during laser–matter interaction in a vacuum [19], air [20] or liquid [21], there exists a minimum fluence that causes the removal of the irradiated material, and each material has a specific value of ablation threshold. The material can be ablated only if the laser fluence is greater than the material’s threshold [22]. In the case of a high-fluence femtosecond laser pulse, the high heat penetration depth causes an increased thermal diffusion, resulting in the ablation of the material by melting and vaporization, even at very short pulse duration. Long pulse duration induces a decrease in ablation efficiency due to heat loss in the target and laser-induced shielding [23]. The ablation rate increases logarithmically with the laser fluence. Moreover, the ablation rate is higher for shorter wavelengths [24] even if a high concentration of structures dispersed in the solvent, absorbing shorter wavelength radiation more than longer wavelength radiation, reduces the ablation rate [25]. The production of ultrapure and reactive nanoparticles [26] in a high-vacuum atmosphere is possible but expensive.

However, ion energies generated during laser ablation in a vacuum differ from those produced in liquid environments. In a vacuum, ions experience minimal collisional damping and can be accelerated by electrostatic fields within the expanding plume, resulting in energies on the order of hundreds of eV. In contrast, during laser–matter interaction in liquids, the plume undergoes strong confinement, rapid thermalization, and intense collisional scattering. As a result, carbon ions generally exhibit kinetic energies reduced by more than an order of magnitude, typically ranging from a few eV to few-tens-of-eV range, depending on laser parameters and liquid properties.

On the other hand, air can be used as the ablation atmosphere; however, the chemistry of the obtained nanoparticles (NPs) will be affected by the quality of the air. Indeed, the interaction between air molecules and nanoparticles induces agglomeration [27].

Liquid, despite the air, seems to be a safer medium. The laser-ablation process has higher efficiency in water than in air, as shown by Patel et al. [28]. It was observed that the best environment to produce a uniform nanoparticle size distribution is water [29].

A physical route for the fabrication of graphene nanosheets (GNS) and graphene quantum dots (GQDs) is the pulsed laser ablation in liquid (PLA) [30].

The selection of laser parameters during the ablation, like the laser wavelength, pulse width, pulse repetition rate, pulse energy, and fluence, can control the size, shape, size distribution, and structure of the obtained nanostructures [31,32].

The application of an electric field during the laser ablation in liquid seems to promote the production of nanoparticles (NPs) with controlled sizes and unique morphology [33].

The present study is addressed to the production of graphene-based nanostructures by laser ablation in distilled water (DI) and in deionized water (DIW), assisted by an electric field of a solid target of graphite.

Considering the hydrophobicity of graphene, the proper choice of liquid is crucial to avoid the agglomeration and restacking of graphene nanosheets. Otherwise, the advantages of single- or multi-layer graphene would be lost. Indeed, in chemical synthesis, surfactants or stabilizers can be added to prevent restacking, while in pulsed laser ablation in liquids (PLA), the liquid itself acts as the stabilizing medium, directly influencing the nucleation, growth, and dispersion of nanoparticles.

Different liquid media that would hinder the stacking of the graphene nanosheets were investigated in the literature [34]. R. M. Altuwirqi [35] reported mixed products of nanosheets and carbon nanoparticles in water, acetone, and liquid nitrogen depending on the laser parameters and the liquid medium.

Ghavidel et al. [36] used acetone to produce high-quality graphene nanosheets without nanoparticles present on the nanosheets due to the high molecular dipole moment of acetone, which increased the repulsive force between graphene layers. Deionized water seems to be a more suitable medium for the production of better-quality GO nanosheets that are large and with the fewest defects compared to the other liquids [37].

The choice of liquid plays a crucial role in determining the properties of the resulting nanostructures. In this study, DI and DIW have been selected as liquids in PLA processing due to their very low ionic content and minimal impurities. DI, obtained by distillation, contains trace residual ions and minerals, while DIW has removed all ions, resulting in slightly different electrical conductivities and electrostatic screening properties. These differences may influence the growth, aggregation, and size distribution of the nanoparticles when an external electric field is applied. The size, dispersion, and surface chemistry of the graphene nanoparticles are critical for their potential applications in catalysis, sensing, and energy-related technologies [38].

In this study, we focused on the comparison between the use of DI and DIW, as they should be more appropriate for a broader application of the produced nanostructures. Moreover, different electric field values have been applied with the aim of tailoring the size and shape of the generated nanoparticles. Despite the previous literature, our approach demonstrates controlled production of graphene-based nanoparticles with tunable size and morphology using an external electric field. These investigations could be useful for the realization of reproducible structures with different sizes, shapes, and aggregations using one configuration and changing only one parameter. The use of water as a medium supports a wide range of applicability of the produced nanostructures, from nanotechnology to biosensors and medicine.

## 2. Materials and Methods

**Figure 1:**
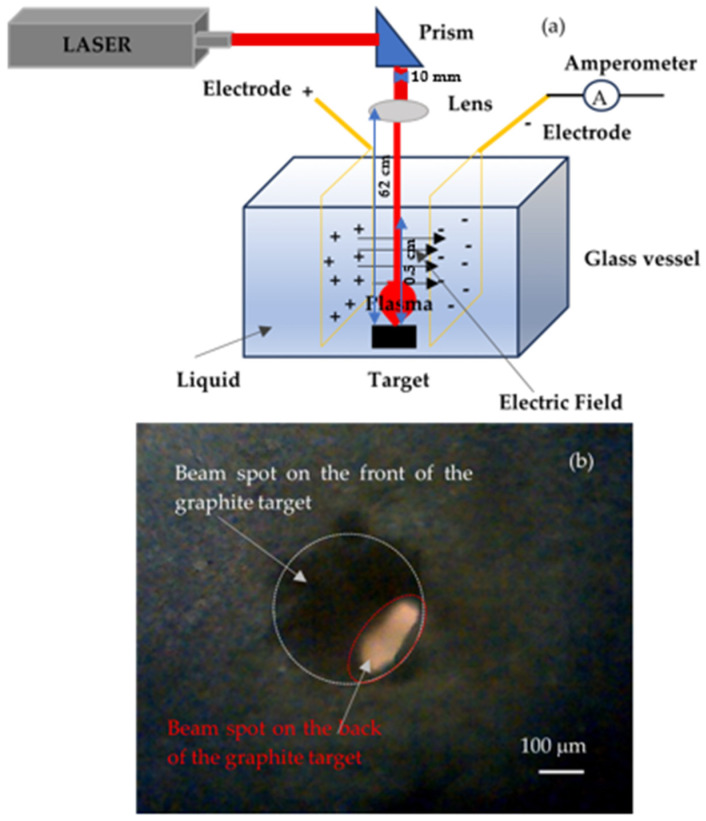
Sketch of the adopted PLA experimental setup, (**a**); optical image of the laser spot on the graphite surface (**b**).

The pulsed laser ablation (PLA) technique was carried out by ablating a solid graphite target submerged in different liquids using a pulsed laser. Typically, the PLA setup is arranged vertically, as shown in Figure 1a. The laser beam is redirected vertically using a prism toward the graphite solid target, which has dimensions of 4 cm × 1.5 cm and a thickness of 2 mm, and is placed at the bottom of a glass vessel filled with liquid.

Two types of water have been used for the preparation of nanostructures by laser ablation of graphite solid targets: DI and DIW. The former is water that has been boiled into vapor and condensed back into water, removing impurities and minerals. The latter is water that has had its ions removed, which means all of the dissolved mineral salts. The water, as a medium, works as an absorbing layer and as a constraining layer.

The PLA experiments were conducted separately in two setups, one using 10 mL of DI and the other using 10 mL of DIW, ensuring that the effects of each liquid could be independently evaluated. Two copper electrodes with 99.99% purity were placed inside the vessel, 3 cm apart and parallel to its sides. The potential difference of 10 V, 20 V, and 50 V between the electrodes created steady electric fields of 10 V/3 cm, 20 V/3 cm, and 50 V/3 cm, while the graphite target was in the middle of them to assist the creation of the uniform electric field during the laser irradiation of the graphite target. The initial 10 mm diameter laser beam was focused by passing through a convex lens placed at a distance of 62 cm from the target surface. The size of the spot on the surface of the target was 300 μm in diameter. This large spot size was selected to operate at moderate laser fluence, ensuring stable ablation conditions and reducing the local heating and the deep crater formation in the graphite target, which could otherwise modify the local laser fluence and ablation regime during irradiation. Moreover, this approach should assist the formation of graphene-based nanostructures rather than large graphite fragments.

In Figure 1b is shown an optical image, 5× magnified, of the size of the hole on the back side of the target, of about 90 μm × 200 μm. The distance between the liquid surface and the target surface is about 0.5 cm. The experimental conditions were fixed, and the ablation medium, DI or DIW, was changed to investigate its effect on fabricated graphene-based nanostructures. The graphite was irradiated under a 1064 nm wavelength, 150 mJ/pulse energy, and 1 Hz repetition rate for an irradiation time of 15 min.

### 2.1. Laser–Matter Interaction in Liquid

The pulsed laser ablation (PLA) in liquid process [39] is a bottom-up approach that can be outlined in four steps: (1) the laser beam hits the target immersed in a liquid; (2) when the ablation takes place, the plasma plume expands differently depending on the surrounding environment (i.e., a stronger confinement in liquid due to higher temperature, pressure, and density); (3) the surrounding liquid is heated to high temperature, inducing vaporization and formation of a liquid plasma; (4) the plasma plume and the liquid plasma are responsible for a chemical reaction between the species of the plume and the liquid plume, leading to the nucleation of atoms to form the nanoparticles.

Graphite consists of a planar stack of graphene layers bonded by weak van der Waals forces. The laser energy released during the PLA process is sufficient to break the weak van der Waals bonds between graphite, producing graphene nanosheets [40]. In the case of laser irradiation in liquid graphite, single carbon atoms can first be detached and then aggregate to form carbon nanoparticles. The interaction between laser and carbon in the liquid can thermalize the system, hindering the melting temperature of carbon, leading to the dispersion of carbon nanoparticles and single or multilayer graphene nanosheets, as observed for the acetone medium [41]. The application of the electric field around the plasma laser generated should control the reactions at the plasma–liquid interface and support the injection of copper ions from the electrode to the plasma for the production of nanoparticles. The characterization of the produced suspensions should prove the cruciality of the applied electric field during the laser ablation to change the morphology and shape of the produced nanoparticles.

### 2.2. Characterization of Graphene Nanostructures

The morphology and size distribution of the produced nanoparticles were assessed through transmission electron microscopy (TEM) analyses. TEM imaging provides direct, high-resolution visualization of nanostructures, essential for validating particle size on the nanometer scale and for correlating morphological features with synthesis conditions. A Jeol 2200 FS (Jeol, Tokyo, Japan) field emission gun (FEG) equipped with an Oxford Instruments EDS analyzer, TVIPS camera, and EM-Menu software (https://www.tvips.com/imaging-software/em-menu/) was employed. The instrument was operated at 200 kV accelerating voltage with a point resolution of 2.4 Å.

However, further analysis with atomic force microscopy (AFM) was carried out on cuts of Si covered with drops of the obtained suspension and dried in air for 24 h. The accurate 2D reconstruction of the sample topography was carried out using a Dimension ICON AFM system (Bruker Corp., Billerica, MA, USA). The SCANASYST in air mode with a spring constant of 0.4 N/m and a scan size of 1 μm^2^ was employed. Images were processed using the NanoScope Analysis 1.80 64-bit software.

The analytical method of UV–Vis spectroscopy was used to measure the absorption between 200 nm and 850 nm wavelength using a JASCO-600 spectrophotometer.

The Attenuated total Reflection Fourier-Transform Infrared (ATR-FTIR) spectra of the suspensions were collected using FTIR spectroscopy, a JASCO 4600 spectrometer, in the range of 4000–400 cm^−1^ on the Si cuts covered with drops of the suspensions dried in air for 24 h.

## 3. Results and Discussion

The laser irradiation of graphite in water generates high-pressure shock due to the expansion of the plasma plume, which reacts with the surrounding liquid both physically and chemically. The formed bubbles expand at high speed, increasing the pressure gradient and then collapsing. The carbon structures (atoms, ions, and clusters) inside the bubble are then dispersed in the liquid. Finally, fragmentation of graphene sheets and the ejection of carbon atoms and ions are expected.

**Figure 2:**
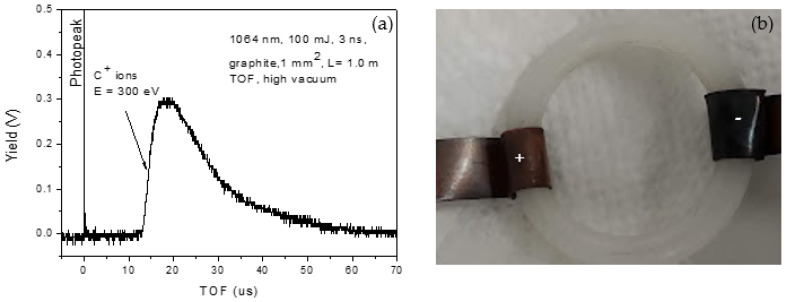
Faraday cup spectrum (**a**); detail of the carbon deposition on the negative electrode (**b**).

Figure 2 shows the spectrum acquired with a Faraday cup (FC) positioned 1 m from a graphite target placed under high vacuum and irradiated with the same laser parameters used for nanoparticle formation in liquid. The detector signal was analyzed by time-of-flight (TOF) [42]. The main peak at ~14 µs corresponds, for the known flight distance, to singly charged carbon ions (C^+^) with an estimated kinetic energy of approximately 300 eV. This spectrum therefore provides direct confirmation that laser–matter interaction under vacuum conditions produces energetic carbon ions, as evidenced by the clear TOF peak attributable to C^+^ species.

**Figure 3:**
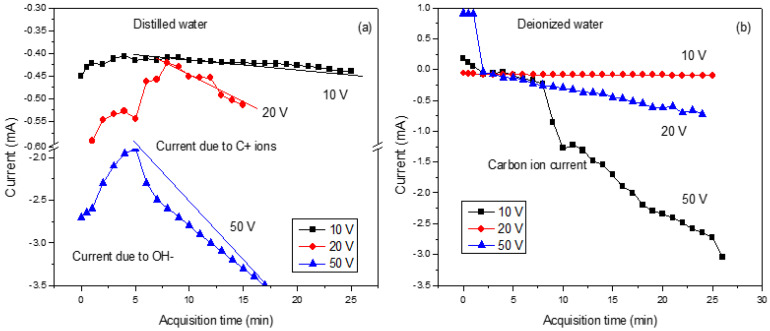
Current vs. time in DI (**a**) and DIW (**b**) for voltage differences of 10 V, 20 V, and 50 V.

In the case of DI, the active OH species formed during the water decomposition react with plasma, forming carbon-based NPs. When the plasma-induced breakdown of water molecules occurs, the synthesized nanoparticles may form oxides or hydroxides. Also, the hydroxyl group can be absorbed on the surface of the ablated nanoparticles, making them electrostatically stabilized. There were reports based on the effect of the temperature of the water on the size of the ablated nanoparticles [43]. Figure 3 shows the trend of the ion current produced during the laser irradiation of the graphite target under the application of a variable electric field.

In Figure 3a, it is clear that the initial decrease in the ions is due to the formation of OH^−^ ions. Then, the increases are due to the contribution of generated C ions with one charge state. On the contrary, in Figure 3b, the measured current increases due to the contribution of ions and salts dispersed in the deionized water. In both cases, the measured current increases progressively as voltages are applied from 10 V to 50 V.

UV–Vis analyses provide insight into the composition of the analyzed suspensions and the concentration and size of contained structures. It is useful to note that carbon materials exhibit two bands: one in the range 260–270 nm, which is assigned to the π–π* transition of the C=C bond, and one in the range 270–350 nm, which corresponds to the n–π* transition of the C=O bond [44,45].

**Figure 4:**
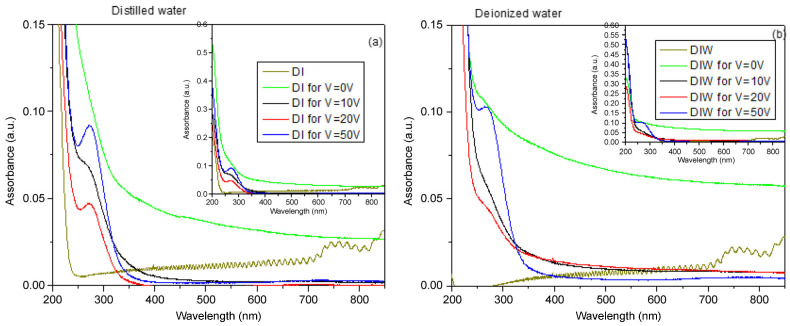
UV–Vis absorbance spectra of graphene-based nanostructures produced in DI (**a**) and in DIW (**b**); changing the applied external voltage.

A negligible absorbance for both DI and DIW water, with a peak in the first case around 200 nm, is presented in Figure 4a,b. DI and DIW refer to pure distilled water and pure deionized water used as reference samples, measured without graphite target, laser irradiation, or electrodes, to obtain the optical baseline of the solvents.

DI for V = 0 V and DIW for V = 0 V correspond to distilled water and deionized water in which the graphite target was immersed and irradiated by the laser, without any applied external voltage. Even though no electric field was generated, pulsed laser ablation occurred, inducing the formation of carbonaceous and graphene-based species in the liquid.

The two suspensions obtained by irradiating the graphite target in DI and DIW for 15 min by laser, without the use of an electric field, revealed absorbance of about 0.05 and 0.08, respectively, while the long tail to the higher wavelengths suggests the inhomogeneous presence of wide-sized structures.

The absorption peak at about 270 nm is more pronounced in DI than in DIW. In Figure 4b, a tail is visible between 400 nm and 800 nm when a voltage of 0 V was applied, indicating structures of large size mixed with structures of smaller size. As the applied electric field increases, this tail becomes less pronounced, suggesting the formation of smaller, more uniformly dispersed structures.

Separately, a weak and narrow absorption feature around 400 nm is not always observed in both distilled and deionized water. This peak is not related to the applied voltage and is not characteristic of intrinsic graphene electronic transitions, which mainly exhibit π–π* and n–π* transitions in the UV region. It is probably due to secondary effects, such as light scattering from a small amount of larger carbonaceous aggregates, and does not influence the size trends discussed above.

**Figure 5:**
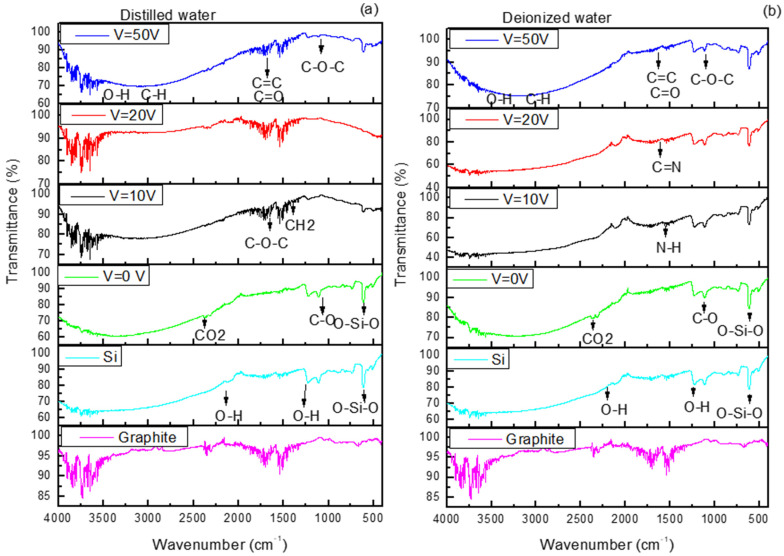
FTIR spectra of suspensions produced in DI (**a**) and DIW (**b**).

Attenuated total Reflection Fourier-Transform Infrared (ATR-FTIR) spectra in Figure 5 show the presence of carbon nanostructures in the 400–4000 cm^−1^ range. In Figure 4a,b, the spectra related to graphite and Si are the same, while the spectra obtained from Si cuts covered with DI and DIW when V = 0 was applied are very similar, indicating the presence of carbon-based structures at 1098 cm^−1^ and at 1633 cm^−1^ ascribable to the stretching vibrations of C-O and to aromatic bending of C=C groups, respectively.

The bands at 1637 cm^−1^ and 1371 cm^−1^ are related to the vibrations of C-O-C bonds and CH_2_, and the band at 1023 cm^−1^ is assigned to C-N. The absorption bands visible in the range 1300–1500 cm^−1^ and 580–780 cm^−1^ are assigned to the bending vibration of C-H from methyl and the stretching vibration of C-H from methylene, respectively [46,47].

In Figure 5b, the peak at 3420 cm^−1^ is ascribed to the O–H stretching vibrational mode, 2930 cm^−1^ is attributed to the C–H stretching, 1633 cm^−1^ is related to aromatic bending of C=C bonds and C=O stretches, and 1068 cm^−1^ is characteristic of C–O–C bonds.

In both Figure 5a,b, FTIR spectra are displayed with characteristic absorption bands of carbon dots, like bending vibrations of N-H at 1570 cm^−1^. The stretching vibrations of C=C and/or C=N (~1600–1610 cm^−1^) bonds confirm the presence of carbon aggregates [48]. The presence of the N contribution may be attributable to the impurities contained in DIW.

**Figure 6:**
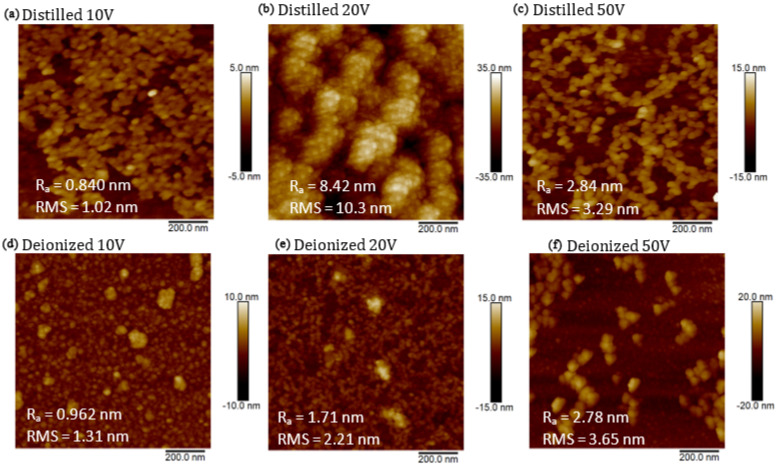
AFM images of suspensions produced by PLA in distilled water (DI) at applied voltages of 10 V (**a**), 20 V (**b**), and 50 V (**c**), and in deionized water (DIW) at applied voltages of 10 V (**d**), 20 V (**e**), and 50 V (**f**).

In Figure 6 are reported the images obtained by the AFM system exploring an area of 1 μm^2^ of Si cuts covered with DI and DIW suspensions containing CDs. Nanoparticles produced in DI at applied voltages of 10 V (see Figure 6a) and 50 V (see Figure 6c), as well as those produced in DIW at 10 V (see Figure 6d) and 50 V (see Figure 6f), exhibit sizes ranging between 10 nm and 30 nm. In DIW, the size of the nanostructures increases with the increase in voltage, while in DI at 20 V (see Figure 6b), we obtained structures with a size of 8 nm. The formation of aggregates does not allow better recognition of the size of the formed nanostructures. A preliminary observation suggests that nanostructures produced in DIW are more stable than those produced in DI, and the assistance of the electric field supports the reduction in nanoparticle size, which, in the absence of an electric field, typically is of about 10 nm in diameter [49].

**Figure 7:**
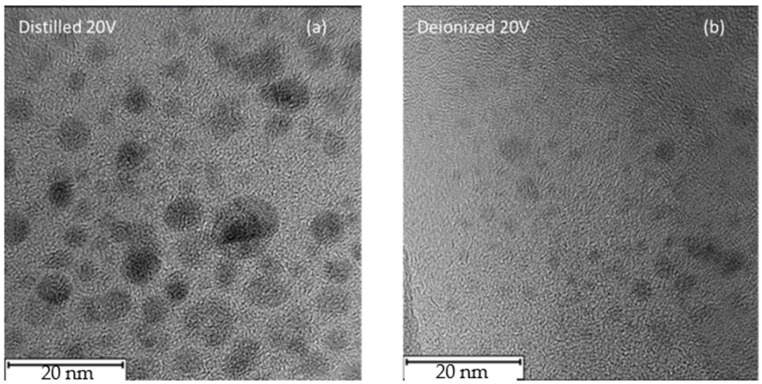
TEM micrographs of suspension produced in DI (**a**) and DIW (**b**).

In Figure 7, high-contrast TEM micrographs of the nanoparticles produced in DI and in DIW when an external potential difference of 20 V is applied are reported. In the first case, the NPs exhibit a mean diameter of approximately 8 nm, showing well-defined, nearly spherical structures. In contrast, when DIW is used, the resulting nanoparticles display significantly smaller dimensions, with an average diameter of about 3 nm.

The size distribution of the laser-generated nanoparticles in both DI and DIW was obtained from transmission electron microscopy (TEM) images using the open-source ImageJ software 2024. TEM micrographs were first imported into ImageJ [50], and the pixel scale was calibrated by drawing a line along the known scale bar on each image. The measured pixel length was then set to the known distance of 0.058 nm/pixel, derived from the TEM scale bar, to convert all measurements into nanometers.

**Figure 8:**
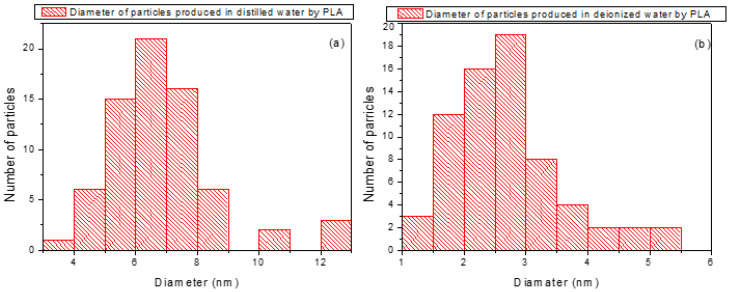
Diameter distributions for nanoparticles synthesized in distilled water (**a**) and deionized water (**b**) by PLA.

To distinguish nanoparticles from the background, contrast adjustments and thresholding were applied. Each nanoparticle identified in the calibrated images was measured, resulting in diameter distributions. For nanoparticles synthesized in distilled water, the distribution revealed particles predominantly in the 5–8 nm range, whereas for those in deionized water, the distribution was centered on 1–3 nm, indicating a clear difference in size distribution between the two media. Histograms of the diameter sizes were plotted in Figure 8a,b to visualize the two distributions.

These TEM observations provide crucial evidence supporting the proposed mechanism of nanoparticle formation and demonstrate the sensitivity of the process to both the physicochemical properties of the liquid and the applied electrical parameters.

The AFM and TEM observations provide crucial evidence that nanoparticle size and stability depend on both the applied voltage and the used liquid. In DIW, nanoparticles remain small (1–3 nm) and well dispersed, whereas in DI, larger structures and aggregation are observed, particularly at 20 V. This behavior could be related to the different ionic contents of the two liquids. DIW, characterized by very low conductivity and long Debye length, provides weaker electrostatic screening, which enhances repulsive interactions between charged carbon clusters and suppresses aggregation, assisting a nucleation-growth regime. In contrast, the higher ionic content of DI is responsible for the stronger electrostatic screening and reduced repulsion under an applied electric field, supporting cluster coalescence and the formation of larger nanoparticles.

Accordingly, TEM images at 20 V reveal significantly smaller nanoparticles in DIW (~3 nm) compared to those produced in DI (~8 nm), demonstrating that nanoparticle formation in PLA is highly sensitive to both the physicochemical properties of the liquid and the applied electrical parameters.

**Figure 9:**
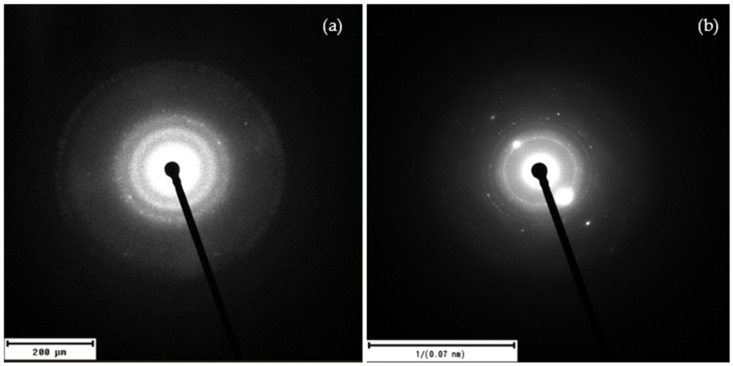
SAED pattern of laser-ablated carbon nanoparticles in DI (**a**) and in DIW (**b**).

Figure 9 shows the patterns of the selected area electron diffraction (SAED) analysis of the laser-ablated carbon nanoparticles produced in DI and in DIW.

The SAED analysis of the laser-ablated carbon nanoparticles reveals, for DI (Figure 9a), well-defined diffraction rings with d-spacings matching the (002), (100), and (110) planes of graphite. This confirms that the laser-ablated carbon structures are polycrystalline graphene/graphite nanosheets. Figure 9b shows the SAED pattern exhibiting sharp diffraction rings with discrete spots, indicating a predominantly polycrystalline structure with regions of larger or preferentially oriented crystallites. The reciprocal space scale marked as “1/(0.07 nm)” means that the scale bar corresponds to a distance in reciprocal space equivalent to lattice planes with interplanar spacings of about 0.07 nm. This notation allows the identification of crystalline planes with very small interplanar distances, consistent with graphitic crystalline structures. The presence of sharp diffraction rings, rather than diffuse halos, suggests a predominantly crystalline structure with minimal amorphous content. High-resolution TEM (HRTEM) studies would be valuable for direct visualization of lattice fringes and confirmation of graphene-like domains, which could be addressed in future work.

The two main mechanisms induced by nanosecond lasers to ablate material are the explosive ejection of molten droplets from the irradiated target, with nanometric/micrometric sizes by thermal effect, and the thermal vaporization of ionic or atomic species from the laser-irradiated target surface.

The two suspensions obtained by laser irradiation of a solid target of graphite in DI and DIW were analyzed by UV–Vis and FTIR. The observed absorption peaks can be attributed to the increase in the number of nanostructures, which should be due to the increased temperature [43,44,45,46,47,48,49,50,51].

In both cases of PLA in distilled and deionized water assisted by an electric field, we observed the formation of carbon nanostructures of different sizes. With the increase in the electric field, in the former, the average size was smaller, and the gradient was more restricted than in DIW. In the optical UV-Vis spectroscopy, the absorption peaks observed around 271 nm can be attributable to π–π* transitions of the C=C bonds within conjugated fragments in the carbon core [52]. The FTIR analysis confirmed the presence of C=C, a C=O, and O-H groups ascribed to the use of graphite and water. The size of the nanoparticles at 50 V external voltage seemed to be more uniform and better dispersed in DI than in DIW. The gradual size increase in DIW and the formation of larger (~8 nm) nanoparticles in DI at 20 V can be explained by the different physicochemical properties of the two liquids. In particular, the ionic strength of the liquid determines the Debye length (λ_D_), which controls the range of electrostatic interactions between charged nanoparticles in colloidal systems. DIW, having very low ionic content, exhibits a relatively long Debye length (λ_D_) and consequently weaker electrostatic screening and higher colloidal stability. This limits particle–particle interactions and assists the formation of smaller nanoparticles. On the contrary, DI contains a slightly higher concentration of residual ions, leading to a shorter Debye length and enhanced electrostatic screening. It was observed that applying 20 V can reduce electrostatic repulsion and enhance the local electric field around the laser plume, and promote the coalescence of carbon clusters, leading to the formation of larger nanoparticles.

Typically, the conductivity of DI ranges from sub-μS/cm up to about 10 μS/cm, depending on impurity level [53]. The literature [33] reports better control of the size and morphology of Pt-NPs as well as an increase in their concentration in the case of an applied external electric field. DIW is typically used as a medium during the production of NPs by laser ablation in liquid, as it has a high heat capacity [54] and a conductivity of 0.056 μS/cm.

## 4. Conclusions

An environmentally friendly approach based on the use of a pulsed laser ablation assisted by an electric field was adopted for the production of graphene-based nanoparticles. The electric field, applied around the plasma laser generated, supported the modulation of the plasma parameters. The variation of the electric field across the Cu electrodes assisted in the control of the size, morphology, and density of the produced nanoparticles. Comparisons between the NPs produced in the presence of an electric field and without an electric field were conducted. AFM was used to investigate the structural, compositional, and morphological characteristics of the produced nanoparticles. The introduction of an electric field during laser ablation changes the size and dispersion of the distributions.

A direct comparison between distilled water and deionized water highlights the key role of the liquid medium in the determination of nanoparticle features, revealing significant differences in size and morphology depending on the liquid used.

Deionized water seems to be a more suitable medium for the production of better-quality carbon nanoparticles, and the assistance of the electric field supports the reduction of particle size, which, in the absence of an electric field, typically is of about 10 nm in diameter.

The pulsed laser ablation approach employed in this work enables the synthesis of high-purity graphene-based nanoparticles without the use of chemical reagents, surfactants, or hazardous compounds, minimizing contamination effects. The capability to tailor nanoparticle properties through the combined control of the liquid environment and the applied electric field, while keeping the laser parameters constant, indicates that the method is flexible and adaptable for further optimization.

However, although the size of the formed graphene-based material was lower than 10 nm, no luminescence property was observed.

This study provides new insight into electric-field-assisted laser ablation in liquids and represents a step forward toward controllable and potentially scalable fabrication routes for graphene-based nanomaterials, with relevance for environmentally sustainable technologies.

## Data Availability

The original contributions presented in this study are included in the article. Further inquiries can be directed to the corresponding author.
